# Mechanism of cell death induced by the novel enzyme-prodrug combination, nitroreductase/CB1954, and identification of synergism with 5-fluorouracil

**DOI:** 10.1038/sj.bjc.6601211

**Published:** 2003-08-26

**Authors:** D H Palmer, A E Milner, D J Kerr, L S Young

**Affiliations:** 1 CR UK Institute for Cancer Studies, Clinical Research Block, University of Birmingham, Birmingham B15 2TA, UK

**Keywords:** VDEPT, nitroreductase, CB1954, apoptosis, synergy, 5-fluorouracil

## Abstract

Virus-directed enzyme prodrug therapy (VDEPT) utilising the bacterial enzyme nitroreductase delivered by a replication-defective adenovirus vector to activate the prodrug CB1954 is a promising strategy currently undergoing clinical trials in patients with a range of cancers. An understanding of the mechanism of tumour cell death induced by activated CB1954 will facilitate this clinical development. Here, we report that activated CB1954 kills cells predominantly by caspase-dependent apoptosis. This may have important implications for the generation of immune-mediated bystander effects. Further, the use of a replication-defective adenovirus vector to deliver nitroreductase may negatively affect cellular apoptotic pathways stimulated by activated CB1954. Finally, examination of nitroreductase/CB1954 in combination with conventional chemotherapy reveals a synergistic interaction with 5-fluorouracil. These data will facilitate the further development and future clinical trial design of this novel therapy.

Epithelial ovarian cancer is the leading cause of death from gynaecological cancer with the majority of patients presenting with advanced intraperitoneal disease and requiring chemotherapy. The current standard treatment is a combination of carboplatin or cisplatin with paclitaxel. However, 5-year survival remains poor at 25–30%. It is imperative therefore that novel treatments are developed.

Chemotherapeutic drugs can kill cells by activating apoptotic pathways and the exact mechanism by which this occurs is currently under intense investigation. Initial studies revealed that cytotoxic drugs can activate death receptor/caspase-8 signalling pathways and this mechanism may be an essential factor in the early phase of drug-induced cell death depending on the cell type used (Friesen *et al*, 1996; Fulda *et al*, 1997, 1998a, 1998b, 2000; Micheau *et al*, 1997). However, not all cell types require death receptor–ligand interaction for drugs to be effective (Gamen *et al*, 1997; Eischen *et al*, 1997; Villunger *et al*, 1997; Tolomeo *et al*, 1998; Ruiz-Ruiz Q1and Lopez-Rivas, 1999). Recent studies have indicated that caspase-8 can be activated independently of death receptor ligation (Milner *et al*, 2002). Interestingly, different drugs may engage distinct components of the apoptotic machinery (Milner *et al*, 2002). This is important since it may allow the use of different drugs to form potentially synergistic combinations.

A novel approach to cancer therapy currently entering clinical trials is Virus-Directed Enzyme Prodrug Therapy (VDEPT). This utilises an adenovirus delivered enzyme, nitroreductase (NR), to catalyse the conversion of a relatively nontoxic prodrug (CB1954) into an extremely potent DNA cross-linking agent. Preclinical studies have shown that adenoviral delivery of NR to a range of human cancer cell lines sensitises them to CB1954 by 500–2000-fold compared to the parental cell line (Weedon *et al*, 2000). Further, in an *in vivo* xenograft model of peritoneal pancreatic cancer (Suit 2), a doubling in median survival was seen in mice treated with virus and CB1954 compared to controls (*P*<0.0001) (Weedon *et al*, 2000).

*In vitro* cell mixing experiments using unmodified and NR-expressing ovarian carcinoma cell lines have demonstrated significant sensitisation (30–100-fold) of the total cell population to CB1954 when only 5–10% of the cells express NR (McNeish *et al*, 1998). Similarly, *in vivo* in a human hepatoma murine xenograft model, a significant antitumour effect and improved survival were observed even when only 5% of cells expressed NR, confirming a significant bystander effect (Djeha *et al*, 2000). Interestingly, studies of other enzyme–prodrug combinations also suggest a ‘distant bystander effect’ thought to be a systemic immune-mediated response to local tumour cell killing (Link *et al*, 1997). Other potential advantages of the NR/CB1954 combination are the ability to kill cells in a cell cycle-independent manner and the lack of cross-resistance with other commonly used cytotoxic agents (Bridgewater *et al*, 1995; Knox, 1998). A recent phase I clinical trial has confirmed that CB1954 is a well-tolerated prodrug that can be administered intravenously or intraperitoneally at doses sufficient for a VDEPT approach to be feasible in the treatment of ovarian cancer (Chung-Faye *et al*, 2001). A further clinical trial to assess the safety, tolerability and transgene expression of a replication-deficient adenovirus vector carrying the gene for nitroreductase is ongoing. It is important, therefore, to investigate the mechanism by which activated CB1954 kills NR-expressing cancer cells. This will facilitate the further clinical development of this system in enhancing distant bystander effects and in devising rational combinations with conventional chemotherapeutic agents.

The aims of the current study are: (1) to determine the mode of cell death initiated by activated CB1954, (2) to identify the apoptotic pathways induced by activated CB1954 and in particular to identify any requirement for caspase activation, and (3) to assess the effect of CB1954 given in combination with a range of other chemotherapeutic agents in order to identify mechanistically synergistic drug combinations that may be utilised in future clinical trials.

## MATERIALS AND METHODS

### Cells and cell culture

The human ovarian carcinoma cell line SKOV3 was maintained in DME with HEPES, 10% FCS and supplemented with glutamine. Stable NR-expressing SKOV3 cells (SKOV3NR) generated by infection with a retrovirus vector carrying the gene for *E. coli* bacterial nitroreductase were the kind gift of Dr PF Searle (University of Birmingham, UK).

### Detection of NR and caspases by Western blotting

Cells were harvested, washed once in ice-cold PBS, pelleted by centrifugation and pellets lysed for 20 min on ice. Samples were pelleted at 13 000 rpm at 4°C and lysates decanted and stored at −70°C. Protein concentrations of total cell lysates were determined using a Biorad (Hercules, CA, USA) assay. Total cell lysate protein (100 *μ*g) was separated by SDS–PAGE and electroblotted onto nitrocellulose. Membranes were blocked with 5% milk in PBS–Tween and then probed with either polyclonal nitroreductase antibody 108B (kindly provided by Dr M Ford, Glaxo-Wellcome, Stevenage, UK) diluted 1 : 20000, or polyclonal rabbit anti-caspase-8 (a kind gift from Dr G Cohen, University of Leicester, UK; Sun *et al*, 1999) diluted 1 : 3000, monoclonal mouse anti-caspase-9 (Calbiochem) diluted 1 : 100, polyclonal rabbit anti-caspase-3 (Pharmingen, San Diego, CA, USA) diluted 1 : 5000. Immune complexes were detected with horseradish peroxidase-conjugated secondary antibodies (anti-rabbit diluted 1 : 2000; anti-mouse diluted 1 : 1000; Sigma, UK) and then visualised with ECL reagent (Amersham, UK) and autoradiography.

### Adenovirus infection of cells

SKOV3 cells were plated out at a density of 2 × 10^6^ cells ml^−1^ in 75 cm^3^ flasks and allowed to attach overnight. The medium was removed and the cells were exposed to either RAd-NR, an E1-, E3-deleted replication-defective adenovirus expressing bacterial NR from the CMV promoter, or a control *β*-galactosidase-expressing adenovirus (RAd35, kindly provided by Dr G Wilkinson, Cardiff, UK; Wilkinson and Akrigg, 1992), in 1 ml of medium, rocking the flask every 20 min. After 2 h the infection medium was removed and replaced with 12 ml of fresh medium. After 48 h, cells were harvested for Western blot analysis of NR expression, or treated with CB1954 prior to analysis of cell death.

### Flow cytometric analysis of cell viability

Cells were plated out at a density of 2 × 10^5^ cells ml^−1^ in 48-well plates and allowed to attach overnight. Following treatment with either CB1954 or chemotherapeutic drugs for 48 h, cells were trypsinised and resuspended in 0.5 ml saline (prewarmed to 37°C). Syto 16 (Molecular Probes Europe, Leiden, Netherlands) was added at a concentration of 25 nM and incubated with the cells at room temperature for one 1 h, at which time 5 *μ*g ml^−1^ propidium iodide was added. Samples were analysed immediately on a Coulter EPICS XL flow cytometer. A two-dimensional dot plot was generated of Syto 16 fluorescence *vs* propidium iodide fluorescence. Syto 16 is only taken up by viable cells and propidium iodide only enters cells whose membranes have become permeablised, therefore this technique distinguishes between viable cells (syto 16 +ve, propidium iodide −ve), apoptotic cells (syto 16 −ve, propidium iodide −ve) and necrotic cells (syto 16 −ve, propidium iodide +ve) (Schuurhuis *et al*, 1997). Data for 10^4^ cells were collected for each sample and prior to data collection cell debris was excluded by setting a gate on a forward *vs* side scatter two-dimensional dot plot.

### Caspase inhibitors

The cell-permeable caspase inhibitors IETD-FMK, LEHD-FMK, DEVD-FMK and ZVAD-FMK (Calbiochem, UK) were added to cells for 1 h prior to treatment with either CB1954 or chemotherapeutic drugs. Initially, a range of concentrations (1–200 *μ*M) was tested and from these experiments a concentration of 50 *μ*M was chosen for experiments in this study.

### MTT assay

Cells were plated out at a density of 5 × 10^4^ cells ml^−1^ in 96-well plates and allowed to attach overnight prior to treatment. Following treatment, 20 *μ*l of 10 mg ml^−1^ MTT (3-(4,4-dimethylthiazol-2-yl)-2,5 -diphenyl tetrazolium bromide) (Sigma) in PBS were added to each well and incubated for 4 h at 37°C, and the formazan crystals formed were dissolved in DMSO. The absorbance was recorded at 550 nm.

### Analysis of the cytotoxic effect of CB1954 in combination with other chemotherapeutic agents

The effect of drug combinations was calculated according to the median effect principle described by Chou and Talalay (1984). Firstly, the dose–response curves for the cytotoxic effects of CB1954 and a panel of other drugs (cisplatin, 5-fluorouracil (5-FU), topotecan, doxorubicin, paclitaxel, gemcitabine) in SKOV3 cells either stably expressing NR, or expressing NR delivered by an adenovirus vector, for each agent singly and in combination were constructed.

The median effect principle of mass action states that


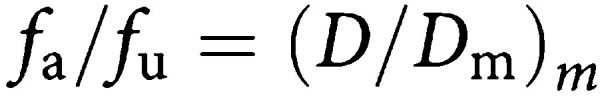


where *D* is the drug dose, *D*_m_ the drug dose required for 50% cell kill, *f*_a_ the cell fraction affected, *f*_u_ the cell fraction unaffected, and *m* the coefficient of sigmoidicity.

This equation can be linearised by taking logarithms of both sides so that *y*=log(*f*_a_/*f*_u_) is plotted with respect to *x*=log(*D*). From this, *m* and *D*_m_ can be derived. Note that the linear correlation coefficient, *r*, describes the conformity of the data to the median effect principle. These data can then be used to determine the ‘combination index’ (CI), using the equation:


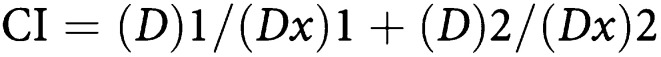


where (*D*)1 and (*D*)2 in combination kill *x*% of cells, and (*Dx*)1 and (*Dx*)2 are the doses of each drug alone that kill *x*% of cells.

If CI <1, =1 or >1, then synergism, additive effect or antagonism is indicated, respectively. So, if the combined observed effect is greater than the calculated additive effect, synergism is indicated.

The validity of this methodology was demonstrated by confirmation of the previously reported synergistic interaction between cisplatin and 5-FU (data not shown) (Shirasaka *et al*, 1993; Tsai *et al*, 1993).

## RESULTS

### The mode of cell death induced by CB1954 is predominantly apoptosis

**Figure 1:**
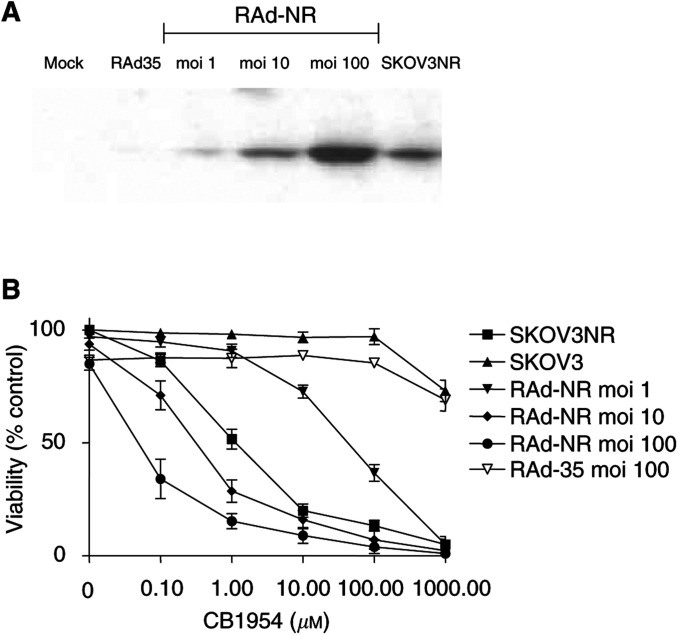
A replication-deficient adenovirus vector carrying the gene encoding bacterial NR results in NR expression and sensitisation to CB1954 cytotoxicity in SKOV3 cells. (**A**) SKOV3 cells were infected with RAd-NR and the presence of NR protein was assessed 48 h later by Western blot analysis. Lane 1, mock infection; lane 2, control virus (RAd-35) moi 100; lane 3, RAd-NR moi 1; lane 4, RAd-NR moi 10; lane 5, RAd-NR moi 100; lane 6, stable NR-expressing SKOV3. (**B**) SKOV3 cells were mock infected, infected with control virus or with RAd-NR (moi 1, 10, 100). After 48 h, cells were treated with CB1954 over a range of doses (0, 0.1, 1, 10, 100 or 1000 *μ*M). SKOV3 cells stably expressing NR were similarly treated. After a further 48 h, cell viability was assessed by MTT assay. Results are expressed as a percentage of the absorbance of untreated cells and are the mean of three separate experiments ±s.e.

The SKOV3 ovarian carcinoma cell line was either selected to stably express NR after retroviral transduction or infected with RAd-NR to produce transient NR expression. A comparison of the levels of NR expression showed that RAd-NR was able to deliver NR in a dose-dependent manner successfully with a virus dose of moi 10 resulting in similar levels of NR expression to that seen in the stably expressing cell line (Figure 1A

). Further, NR sensitised SKOV3 cells to the prodrug CB1954 as measured by MTT assay, the amount of cell death correlating with the level of NR expression (Figure 1B).

**Figure 2:**
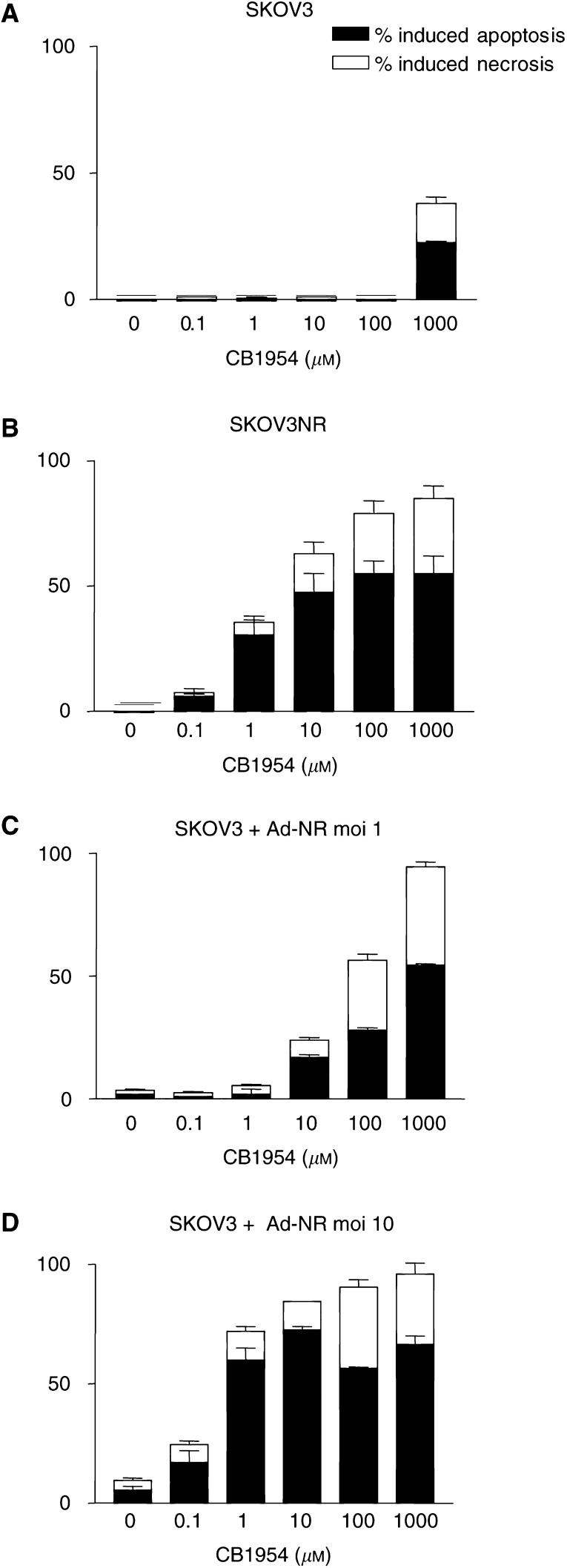
Analysis of the mode of cell death induced by activated CB1954. SKOV3 cells were infected with RAd-NR and then treated with CB1954 as described in Figure 1. The proportion of apoptotic and necrotic cell death was determined by FACS analysis. Results are the mean of three separate experiments ±s.e. (**A**) Parental SKOV3, no NR expression; (**B**) SKOV3 cells stably expressing NR; (**C**) RAd-NR moi 1; (**D**) RAd-NR moi 10.

Parental SKOV3 cells and SKOV3NR cells were treated with CB1954 over a range of concentrations. After 48 h, FACS analysis was performed to determine the degree of cell death induced and the relative amounts of apoptosis and necrosis. Figure 2B

demonstrates that the mode of cell death induced by activated CB1954 in this setting was predominantly apoptosis, in common with a range of other chemotherapeutic agents (data not shown and Milner *et al*, 2002). In addition, RAd-NR infected SKOV3 cells were treated with CB1954 and similar analysis was performed. This confirmed that apoptosis was the predominant form of cell death induced and that the degree of apoptosis increased in proportion with increasing NR expression and increasing CB1954 dose (Figure 2C and D). In the absence of NR, CB1954 was toxic only at very high concentration (Figure 2A).

### Activated CB1954 initiates activation of caspase-8, -9 and -3

**Figure 3:**
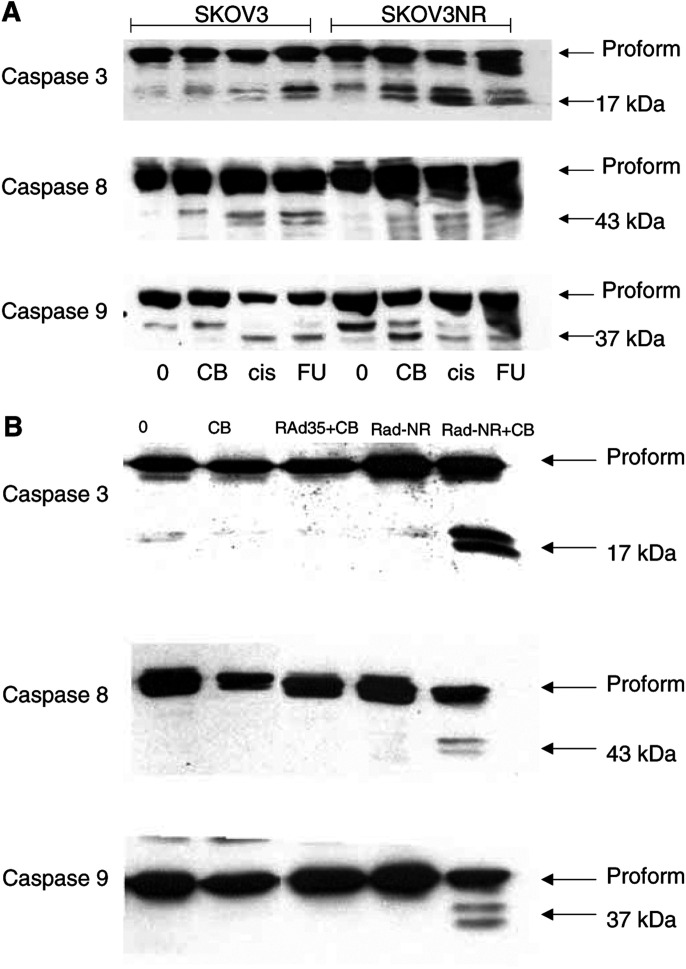
Caspase cleavage by activated CB1954. (**A**) SKOV3 and stable NR expressing SKOV3 cells were treated with CB1954, cisplatin or 5-fluorouracil at doses sufficient to cause apoptosis in approximately 70% of cells (confirmed on samples prepared in parallel and analysed by FACS, data not shown). The dose of CB1954 used to treat parental SKOV3 cells was that which induced 70% apoptosis in NR-expressing cells. At this dose, no apoptosis was observed in the parental cells. After 48 h, the activation of caspase-3, -8 and -9 was assessed by Western blot analysis. Antibodies able to recognise both proform and active form of each caspase were used. Lanes 1–4, parental SKOV3 cells; lanes 5–8, stable NR expressing cells. Lanes 1 and 5, no drug; lanes 2 and 6, CB1954 10 *μ*M; lanes 3 and 7, cisplatin 10 *μ*M; lanes 4 and 8, 5-FU 500 *μ*M. (**B**) SKOV3 cells were infected with RAd-NR (moi 10) or control adenovirus, RAd-35 (moi 10). After 48 h, cells were treated with CB1954 10 *μ*M. After a further 48 h, caspase cleavage was assessed as in (**A**). Lane 1, untreated control; lane 2, CB1954 alone; lane 3, RAd-35+CB1954; lane 4, RAd-NR alone; lane 5, RAd-NR+CB1954.

Stably expressing SKOV3NR cells were treated with a panel of drugs commonly used in the treatment of ovarian cancer and with CB1954 at doses sufficient to induce apoptosis in approximately 70% of the cells. Western blot analysis for caspase-8, -9, and -3 revealed that the active form of each caspase could be clearly detected following treatment with all drugs including activated CB1954 (Figure 3A

). However, in the absence of NR, CB1954 was unable to initiate apoptosis or activate any of these caspases (Figure 3A).

SKOV3 cells transiently expressing NR were also treated with CB1954 to induce high levels of apoptosis and similar Western blots were performed. Again, activation of caspase-8, -9, and -3 was seen. RAd-NR infection alone or CB1954 treatment of cells infected with a control adenovirus vector did not result in apoptosis or caspase activation (Figure 3B).

### The relative contribution of caspases to the induction of apoptosis differs between RAd-NR infected and stable NR-expressing cells treated with CB1954, and between CB1954 and other chemotherapeutic agents

**Figure 4:**
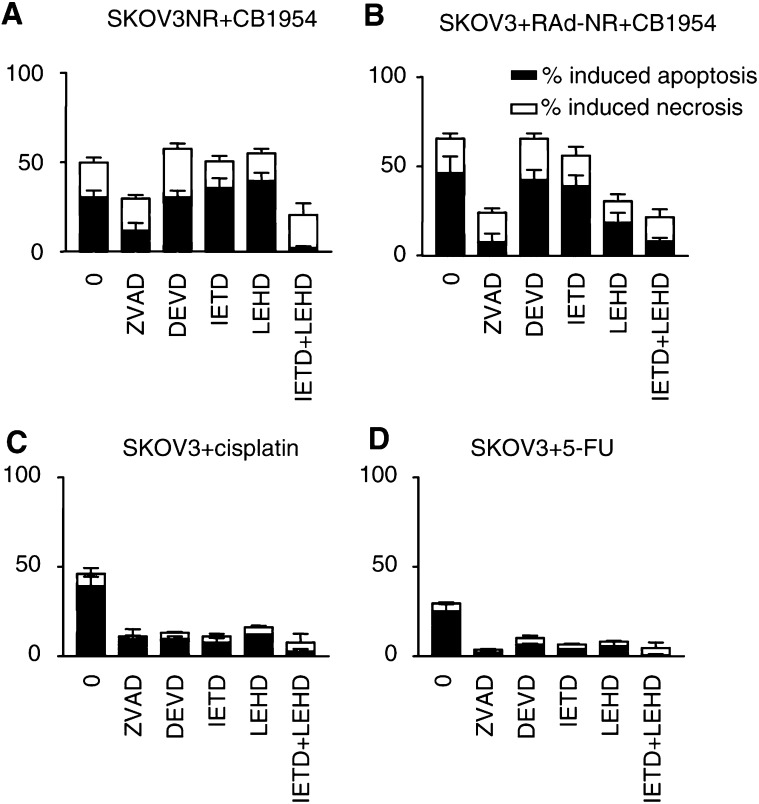
Effect of caspase inhibitors on CB1954-induced cell death. SKOV3 cells stably expressing NR (**A**), or infected with RAd-NR moi 10 (**B**), were treated with CB1954 10 *μ*M in the presence or absence of a broad-spectrum caspase inhibitor (ZVAD-FMK, 50 *μ*M), a caspase-3 like inhibitor (DEVD-FMK, 50 *μ*M), a caspase-8-like inhibitor (IETD-FMK, 50 *μ*M) or a caspase-9 like inhibitor (LEHD-FMK, 50 *μ*M). SKOV3 cells were treated with cisplatin (10 *μ*M) or 5-FU (500 *μ*M) also in the presence or absence of caspase inhibitors (**C**) and (**D**). The levels of cell death were analysed 48 h later by FACS. Results are the mean of three independent experiments ±s.e.

To determine whether the caspase activation seen in Figure 3 is essential for the mechanism of CB1954 induced cell death, stably expressing SKOV3NR cells were treated with CB1954 or one of a range of other drugs either in the presence or absence of specific inhibitors of caspase-3, -8, or -9, or a broad-spectrum caspase inhibitor, ZVAD-FMK. We have previously shown that at a concentration of 50 *μ*M, DEVD-FMK (caspase-3 inhibitor), IETD-FMK (caspase-8 inhibitor) and LEHD-FMK (caspase-9 inhibitor) work in a specific manner (Milner *et al*, 2002). Therefore, this concentration was used for all the inhibitors in these experiments. Figure 4

shows the levels of cell death induced at 48 h by either drugs alone or when cells were preincubated with the inhibitors for 1 h prior to drug treatment.

Pretreatment with the broad-spectrum ZVAD inhibited apoptosis induced by activated CB1954 or by the other drugs used, confirming that apoptosis was caspase-dependent. In addition, pretreatment with each of the specific caspase inhibitors alone resulted in a significant reduction in the level of apoptosis induced by all the conventional cytotoxic drugs (Figure 4C, D and data not shown). However, pretreatment of stably expressing SKOV3NR cells with individual inhibitors alone was not sufficient to decrease CB1954-induced apoptosis, although this was significantly reduced by a combination of caspase-8 and -9 inhibitors. Apoptosis induced by CB1954 after RAd-NR infection was again inhibited by ZVAD. However, in this setting, the specific caspase-9 inhibitor (but not the caspase-8 inhibitor) also resulted in a partial reduction in the level of apoptosis, although a more profound inhibition resulted from combined pretreatment with caspase-8 and -9 inhibitors.

### Combination treatment with CB1954 plus 5-FU causes enhanced tumour cell kill

As the above experiments indicated that apoptotic pathways and, specifically, the relative contribution of caspases activated by CB1954 differ from those initiated by other cytotoxic agents, it was of interest to determine whether combining CB1954 with any of these agents could cause an increase in cell death.

**Figure 5:**
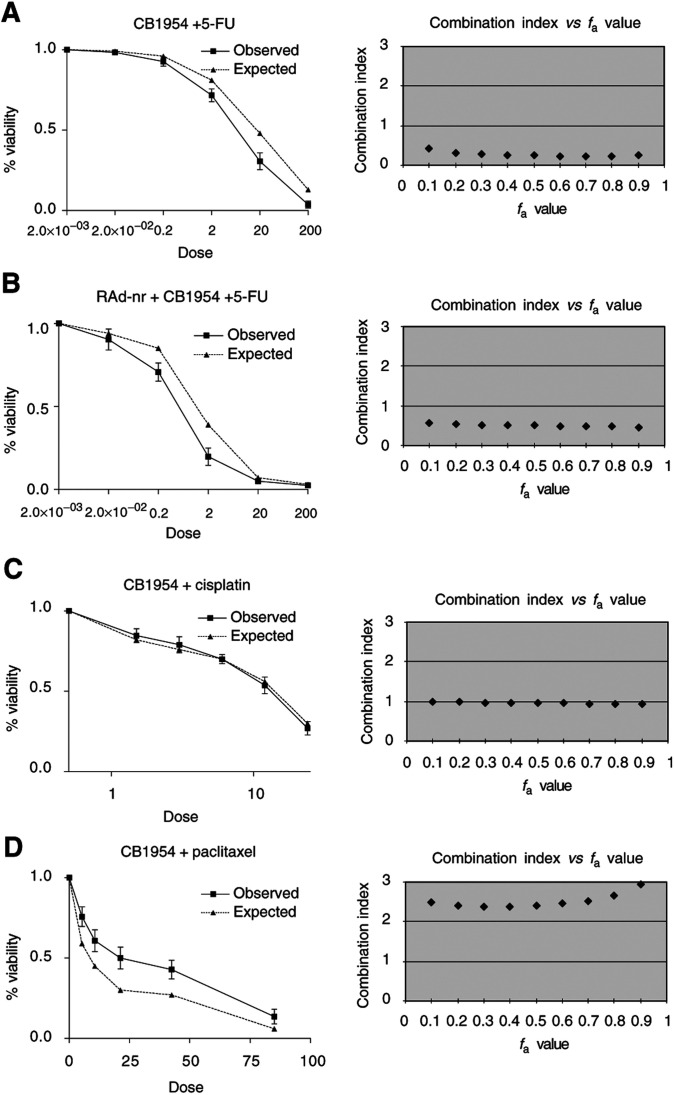
Analysis of cytotoxicity induced by CB1954 in combination with other chemotherapeutic agents. Cell viability in response to treatment with drug combinations was assessed by MTT assay and results expressed as a percentage of the absorbance of untreated cells (solid line; mean of three experiments ±s.e.). The expected effect of each drug combination based on an additive interaction was calculated (broken line). Therefore, synergistic interactions will be to the left and antagonistic interactions to the right of calculated curve. (**A**) Stable NR expressing SKOV3 cells were treated with CB1954 over a range of doses (0–1000 *μ*M), in combination with 5-FU in a fixed dose ratio (1 : 100) according to the median effect principle of mass action. After 48 h, cell viability was assessed by MTT. (**C**) CB1954 in combination with cisplatin (dose ratio 1 : 1). (**D**) CB1954 in combination with paclitaxel (dose ratio 10 : 1). (**B**) SKOV3 cells were infected with RAd-NR (moi 1) and 48 h later treated as in (**A**). All results are the mean of three independent experiments ±s.e. The combination index is also shown for each drug combination, where a value of 1=additive, <1=synergistic and >1=antagonistic.

Stably expressing SKOV3NR cells were treated with CB1954 simultaneously with one of 5-FU, cisplatin, topotecan, doxorubicin, paclitaxel or gemcitabine over a range of drug concentrations. After 48 h, cell viability was assessed using the MTT assay. These data were then used to calculate ‘CI’ plots to assess whether drug combinations were synergistic, additive or antagonistic. It was demonstrated that CB1954 in combination with 5-FU produced a synergistic enhancement of cell killing over a wide range of drug concentrations in both SKOV3NR cells and SKOV3 cells infected with RAd-NR. Of note, in the absence of NR, CB1954 did not potentiate 5-FU cytotoxicity. The addition of cisplatin, topotecan, doxorubicin, paclitaxel or gemcitabine to CB1954 resulted in, at best, additive cell killing and in some cases was even antagonistic (Figure 5

and data not shown). Similar results, specifically demonstrating synergy between activated CB1954 and 5-FU, have been obtained in human colon cancer cell lines (WiDr and SW480, data not shown).

## DISCUSSION

In this study, we have investigated the mechanism of cell death induced by the VDEPT combination of NR and CB1954 in ovarian carcinoma cells and have demonstrated a synergistic interaction between activated CB1954 and 5-FU.

In common with a range of cytotoxic drugs with diverse mechanisms of action, cell death induced by activated CB1954 was predominantly apoptotic rather than necrotic. This distinction is important as it is thought that apoptotic cell death may not be optimal in generating immune responses to tumour antigens, which might enhance tumour cell killing *in vivo* (Melcher *et al*, 1999; Steinman *et al*, 2000). So, immune-mediated bystander killing *in vivo* may be enhanced by combined NR/CB1954 gene therapy with immunostimulatory molecules such as GM-CSF (Green *et al*, 2003).

Apoptosis induced by activated CB1954 was dependent upon caspase activation as indicated by the effect of the broad-spectrum caspase inhibitor, ZVAD. However, the relative contribution of caspases to CB1954-induced apoptosis differed from other cytotoxic drugs. Thus, inhibition of either caspase-3, -8 or -9 alone was effective in reducing levels of apoptosis induced by all the conventional cytotoxic drugs tested suggesting that these are critical effectors of apoptosis. However, when CB1954 was activated in cells stably expressing NR, although caspase-3, -8 and -9 were cleaved, inhibition of either one alone was not sufficient to block apoptosis. Inhibition of caspase-8 and -9 together did significantly reduce apoptosis. This indicates that activated CB1954 may activate caspase-8 and -9 separately, either one of which is sufficient for apoptosis, rather than caspase-9 cleavage occurring downstream of caspase-8 via bid cleavage, as reported for other apoptotic stimuli. This is consistent with previous reports indicating that deficiency of certain caspases can result in compensatory activation of alternative caspase pathways, suggesting a degree of redundancy in death activating pathways (Zheng *et al*, 2000).

Interestingly, a different pattern was observed when NR was delivered via an adenovirus vector, with caspase-9 inhibition resulting in a partial reduction in apoptosis. This may be explained by the effect of the adenovirus vector upon antiapoptotic cellular pathways. Indeed, we have demonstrated that such vectors activate the NF-*κ*B pathway which in turn upregulates expression of antiapoptotic cellular pathways (manuscript submitted). Other studies have reported that NF-*κ*B inducible factors can act by inhibiting caspase-8 activity downstream of its cleavage to the active form (Wang *et al*, 1998; You *et al*, 2001). Therefore, the adenovirus vector may activate cellular pathways resulting in caspase-8 inhibition, so that the additional inhibition of caspase-9 may reduce CB1954-induced apoptosis. These effects may be important, since adenovirus delivery of prodrug-activating enzymes or other cytotoxic agents is a major focus of cancer gene therapy protocols. These data will prove useful in the further development of this novel therapy and will facilitate the rational study of NR/CB1954 in combination with other novel anticancer strategies or with conventional chemotherapy.

The current study has addressed the effect of CB1954 given in combination with other drugs to investigate whether potentially synergistic combinations could be identified.

Using the median effect principle of mass action, it was possible to calculate the expected effect of a combination of drugs based on knowledge of their individual effects. This was then compared to the observed effect achieved by the drug combination. The ratio of the expected to the observed effect is the ‘CI’ so that CI<1 indicates a synergistic interaction, CI=1 indicates an additive effect and CI>1 indicates antagonism. CB1954 treatment of SKOV3 cells stably expressing NR in combination with cisplatin, topotecan, doxorubicin, paclitaxel or gemcitabine demonstrated at best additive and in some cases even antagonistic interaction. Conversely, the combination of CB1954 with 5-FU was found to be synergistic across a wide range of doses. This effect was observed whether NR was expressed stably or delivered via an adenovirus vector. These data suggest that, if VDEPT is to be used in combination with conventional chemotherapy, the clinical outcome may be critically dependent upon the drugs chosen. As early phase clinical trials of other VDEPT systems or conditionally replicating adenoviruses in combination with conventional chemotherapy have reported this to be a safe, feasible approach with promising evidence of efficacy, the data presented here will prove useful in the rational design of future clinical trials of VDEPT using NR/CB1954 (Khuri *et al*, 2000).

It will also be important to carry out further research to determine why NR/CB1954 can synergise with some drugs, but have an inhibitory effect with others. Since there are differences in the pathways upstream of caspase cleavage activated by drugs with different intracellular targets, this may determine the interaction of drug combinations. Alternatively, synergy may be related to effects on cell cycle progression (given that 5-FU is a cell cycle-specific drug), effects on mechanisms of drug resistance, interference with activation/inactivation of active metabolites, effects on DNA repair mechanisms or other undefined mechanisms.
